# Prevalence and determinants of cardiac arrhythmias and conduction anomalies in adults aged ≥ 40 years in Jimma Town, Southwest of Ethiopia: a cross-sectional study

**DOI:** 10.4314/ahs.v22i2.27

**Published:** 2022-06

**Authors:** Iyasu Tadesse Bukata, Elsa Tegene, Teshome Gobena, Yohannes Markos Woldesenbet

**Affiliations:** 1 Lecturer of medical physiology, Department of Medical Physiology, Faculty of medical sciences, Institute of Health, Jimma University, Jimma, Ethiopia: eyucatad@gmail.com, phone: +251912492329; P.O.Box: 378, Jimma, Ethiopia; 2 Assistant professor of internal medicine, Department of internal medicine, Faculty of medical sciences, Institute of Health, Jimma University, Jimma, Ethiopia: elsa_teg@yahoo.com, phone: +251911769176; P.O.Box: 378, Jimma, Ethiopia; 3 Assistant professor of medical physiology, Department of Medical Physiology, Faculty of medical sciences, Institute of Health, Jimma University, Jimma, Ethiopia: teshomeglemu@gmail.com, phone: +251911487658; P.O.Box: 378, Jimma, Ethiopia; 4 Lecturer of medical physiology, School of Medicine, College of Medicine and Health Sciences, Wolaita Sodo University, WolaitaSodo, Ethiopia: yohannestry1@gmail.com, phone: +251911016539; P.O.Box: 138, WolaitaSodo, Ethiopia

**Keywords:** Cardiac arrhythmia, prevalence, pisk-factors, 12-lead ECG, Jimma Town

## Abstract

**Background:**

The prevalence of cardiac arrhythmia (CA) in the Ethiopian population is unknown. A community study was conducted to assess the magnitude and predictors of CAs in adults aged≥40 years in Jimma Town.

**Methods:**

A community-based cross-sectional study was conducted in Jimma town from May to July 2017. A total of 634 adults aged 40 years or older were selected using a systematic sampling technique from six kebeles of the Town. Study participants were screened for CA using a 12-lead ECG machine. Face-to-face interviews, anthropometric, important clinical measurements were performed. Data analysis was done using SPSS for windows version 21.0.

**Results:**

A total of 634 study participants, significant CA occurred in 217 individuals (34.2%). Conduction abnormalities and sinus bradycardia were the commonest findings (25.4%). Premature beats (ventricular 1.9%, atrial 1.1%) and atrioventricular nodal reentrant tachycardia (2.1%) were the next most frequent arrhythmias. Arrhythmias were independently associated with smoking(AOR=1.9;P=.047), hypertension(AOR=1.5;P=.02), heart failure(AOR=2.06;P=.023), prior stroke(AOR=4.9;P=.001), previous history of MI(AOR=1.78;P=.039), vigorous intensity activities(AOR=0.56;P=.024), solidified vegetable oil consumption(AOR=3.5;P=.004), and occupation(pensioner, none)[AOR=1.7;P=.017].

**Conclusions:**

CA is highly prevalent in Jimma. Hypertension and history of heart diseases are the most potent predictors of cardiac arrhythmia. Large-scale screening for early detection of arrhythmia has important implications for treatment.

## Background

Chronic non-communicable diseases (CNCDs) have become the leading causes of death and disability worldwide^1^, and cardiovascular diseases (CVDs) are the number one cause of mortality worldwide and places a high medical and socioeconomic burden on developing countries. Rapid urbanization and the demographic shift play a great role in the burden of the disease in these worlds^2^. About half of all cardiac death cases are attributable to sudden cardiac death, defined as natural death from cardiac causes, heralded by abrupt loss of consciousness within 1 hour of the onset of an acute change in cardiovascular status with, or without preexisting heart disease^3^. Among the causes of sudden cardiac deaths, cardiac arrhythmias (CA) confer a substantial risk of mortality and morbidity and this represents a major health care burden worldwide^4^.

Recent years have witnessed important advances in our understanding of the electrophysiologic mechanisms underlying the development of CA. They are generally divided into 2 major categories: 1. enhanced or abnormal impulse formation (ie, focal activity) and 2. conduction disturbances (ie, reentry)^5^, ^6^. Also, one study says that irregular cardiac rhythmic patterns are caused by abnormal initiation or propagation of electrical excitation signals within the heart and leads to cardiac arrest^8^.

There are various types of CA, including atrial fibrillation, conduction disorders, bradycardia, premature contraction, tachycardia, and ventricular fibrillation or fluttering^9^. Studies confirm that primary prevention and early intervention on the potential risk factors of cardiovascular diseases including asymptomatic CAs in the general community is helpful to lessen the morbidity and mortality that will result^10^. For this reason, it would be useful to have reliable data on the prevalence and associated factors of CA in asymptomatic participants in the general population. Despite this, community-based data on the prevalence of cardiac arrhythmias in developing countries like Ethiopia are scarce.

The prevalence of CA in the community is very high. A study from Hong Kong found a 14.9% prevalence^11^ whereas a study conducted by Gogolashvili et al. found a 16.8% prevalence among the rural population of Krasnoyarsk Territory^12^. A cross-sectional study conducted among heavy vehicle drivers found a prevalence of 59.1 %^13^.

On the contrary, a study conducted in Ontario, Canada, among chiropractic patients found 26% premature ventricular contractions (PVCs), 9.6% premature atrial contractions (PACs), 6.4% had atrial fibrillation (AF), 3.2% had missed beats and 3.2% had a bundle branch block^14^. Although the prevalence of CA increases with age, several lines of evidence suggest that sex, tobacco smoking, khat chewing, diabetes, obesity, hypertension, obstructive sleep apnea, congestive heart failure, and stroke have been reported to cause cardiac arrhythmia^15^–^24^. Yet, the prevalence and predictors of CA in the adult Jimma community are not known.

We recently studied the magnitude and predictors of atrial fibrillation in adults aged 40 and above in Jimma town, Southwest Ethiopia, and made the findings available for the scientific community in IJC Heart and Vasculature. The current work is the continuation of that work (Elsah Tegene et al 2019) and we used the same methodology for both of these studies.

## Materials and Methods

### Study Setting, Design, and Sampling

A community-based cross-sectional study was conducted in Jimma town, Southwest Ethiopia. We followed the methods of Elsah Tegene et al 2019 25. Jimma city consists of 17 kebeles (the lowest administrative unit) in which more than 600,000 people dwell. The study was conducted from May to July 2017.

Residents of Jimma town whose age is ≥40 years were included in the study. The sample size was determined using single population proportion formula. A P value of 0.5, the margin of error 0.05, a design effect of 1.5and a non-response rate of 10% were considered. After, extrapolated from world health organization (WHO) estimates for risk factor assessment studies, we got a sample size of 634.

Patients were sampled by the multistage sampling technique. First, six Kebeles were selected from the seventeen Kebeles of the town by simple random sampling, then the sample size was distributed to the six Kebeles employing proportional to size allocation to the Kebeles. The households from the kebeles were selected by the systematic sampling technique. The lottery method was used to sample an individual from the household when two or more adults aged 40 and above were found in the household.

Jimma University Institutional Review Board approved the study. Informed consent was obtained from all participants.

### Data Collection and measurements

#### Medical history of the patients

Demographic information; past and current smoking and drinking habits; history of hypertension; coronary heart disease; diabetes mellitus and stroke; the prevalence of these diseases in first-degree relatives; use of medications and dietary habits were collected using the questionnaire.

#### Anthropometric measurement

Height was measured using a stadiometer installed with a weight scale and weight was recorded after measuring the participants barefoot to the nearest 0.1 kg using a weight balance (TANITA 380, Tokyo, Japan). participants faced away from the wall with the heels together and the back as straight as possible. The head, shoulders, buttocks, and heels touched the vertical stand. The participant steps away from the wall and the height measurement is recorded to the nearest 0.1 cm. Body mass index was calculated from height and weight measurement.

Waist circumference was measured in accordance with the guideline of the International Diabetic Federation^26^ and we used the European cut-off to interpret the waist circumference measurements for the Sub-Saharan African.

#### Blood pressure measurement

Blood pressure (BP) was measured all three times in the left arm with a digital sphygmomanometer (NUTEC, BP09, CDC). Three seated BP measurements were taken for each subject spaced five minutes apart. The BP was taken using a mercuric sphygmomanometer from the left upper arm after the participant was seated quietly for at least 5 min (average of three measurements). BP was measured using a standard adult arm cuff of aneroid type and a digital sphygmomanometer after 5 min rest. Three readings were taken with 5 min intervals and the average of the three readings was recorded as the final BP of the patient^27^.

### Electrocardiographic examination

A resting 12-lead body surface portable Electrocardiogram(ECG1200G, YSIP-155, Beijing, China) was recorded using regularity of 1 mV = 10 mm and a paper speed of 25 mm/sec and then reviewed by a cardiologist. Electrocardiogram (ECG) electrode explores electrical impulse generated through the extracellular fluid; we first have participants lie supine on examination bed calmly and then clean the subsequent anatomical location for each electrode with alcohol, cut if non-conductive tissue such as hair(in male participants) were there and then applied cardio- cream to enhance conductivity; electrodes were placed accordingly and participants asked to close his/her eyes to avoid disturbances and eventually after clear waveform recruited the electrocardiogram result was printed. Abnormal ECG findings were classified according to the Minnesota Code Classification System: Arrhythmias were classified as premature atrial, nodal, or ventricular beats (code 8.1), ventricular tachycardia (code 8.2), atrial fibrillation or flutter (code 8.3), supraventricular tachycardia (code 8.4), first-degree heart block (code 6.3), left bundle branch block (code 7.1), and right bundle branch block (code 7.2) 28.

### Statistical Analysis

Data were entered into EPI data manager version 4.0.2(The EpiData Association, Odense, Denmark) and exported to SPSS V 21 (SPSS, Chicago, IL, USA) for analysis. Frequency distributions were first explored by cross-tabulations by arrhythmia and were expressed in percentage (%). Continuous variables were expressed as mean ± standard deviation values. Binary and multivariable logistic analyses were applied to assess predictors of CA. Normality of continuous variables was checked using graphic methods (Histograms with normality curves and QQ-plots) and multicollinearity was checked and Hosmer Lemeshow test was done for assessing goodness of model fitness. A p-value ≤ was considered for statistical significance.

## Results

### Socio-demographic characteristics of the participants

A total of 634 participants took part in this study out of whom 360 (56.8%) were females while 274 (43.2%) were males.

The mean age of the participants was 63.3±11.9 years. And the majority of study participants were in the age group of sixty to sixty-nine (60–69).

Around 257(40.5%) were illiterate, 231(36.4%) completed primary level education (1–8), whereas a total of 98(15.5%) completed secondary level education but only 48(7.6%) had tertiary level education (Table 1).

**Table 1:** Distributions of socio-demographic baseline of study participants by arrhythmia status, Jimma Town, May–July, 2017

Variables	Categories	N(%)	Arrhythmia status		% difference	P-value

			Yes (%)	No (%)		
**Sex**	Female	360(56.8)	109(30.3)	251(69.7)	0	0.016
	Male	274 (43.2)	108(39.4)	166(60.6)	9.1%	
Age	40–49	78(12.3)	20(25.6)	58(74.4)	0	0.001
	50–59	133(21.0)	34(25.6)	99(74.4)	0	
	60–69	199(31.4)	66(33.2)	133(66.8)	7.6%	
	70–79	166(26.2)	67(40.4)	99(59.6)	14.8%	
	80+	58(9.1)	30(51.7)	28(48.3)	26.1%	
Ethnic Group	Oromo	295(46.5)	99(33.6)	196(66.4)	2.8%	0.53
	Amhara	108(17.0)	35(32.4)	73(67.6)	1.6%	
	Others*	231(36.4)	83()	148()	13.3%	
Religion	Orthodox		120(36.7)	207(63.3)	5.8%	
	Protestant		10(35.7)	18(64.3)	4.8%	0.47
	Muslim		84(30.9)	188(69.1)	0	
	°Others		3(42.9)	4(57.1)	12%	
Income	Low income		170(32.8)	348(67.2)	0	
	High income		22(43.1)	29(56.9)	10.3%	0.137
Marital Status	Married		119(33.9)	232(66.1)	0.6%	
	Widowed/wer		72(35.1)	133(64.9)	1.8%	
	**Others		26(33.3)	52(66.7)	0	0.943
Occupation	Employee		63(29.7)	149(70.3)	3.3%	0.000
	House wife		55(26.4)	153(73.6)	0	
	^Others		99(46.3)	115(53.7)	19.9%	
Educational Status	Illiterate		83(32.3)	174(67.7)	0	0.468
	Primary		81(35.1)	150(64.9)	2.8%	
	2^ndary^Educ.		32(32.7)	66(67.3)	0.4%	
	Diploma +		21(43.8)	27(56.3)	11.5%	

* Wolayta, Kafa, Dawuro, Gurage, Silte, Yem etc.

° Catholic, J. witness, Waqefata

** single/unmarried, separated, Divorced

^ None, Pensioners.

### Prevalence of cardiac arrhythmia

The prevalence of arrhythmias was 217 (34.2%). Left bundle branch block found in 50(7.9%) but right bundle branch block found in 35 (5.5%) individuals. Sinus bradycardia was found in 48(7.6%) whereas sinus tachycardia was seen in 32 (5.0%) of study participants. Atrial fibrillation was seen in 27(4.3%) individuals. First-degree, second-degree, and third-degree atrioventricular blocks were seen in 1.6, 0.6, and 0.2, respectively (See Table 2 below). Arrhythmia prevalence increased with age. Those whose age eighty and above were 26.1% more prone to develop arrhythmia than those who were in their forties (Fig 1).

**Table 2:** Prevalence of types of cardiac arrhythmias from ECG findings, Jimma town, May to July, 2017

Cardiac Arrhythmias	Frequency(n)	Percent (%)
Conduction disturbances		
Sinus bradycardia	48	7.6
1^rst^ degree AVB	7	1.1
2^nd^ degree AVB	4	0.6
3^rd^ degree AVB	1	0.2
Complete LBBB	50	7.9
Incomplete LBBB	11	1.7
Complete RBBB	35	5.5
Incomplete RBBB	5	0.8
Sinus tachycardia	32	5.0
Sinus arrhythmia	6	0.9
Atrial flutter	3	0.5
Atrial fibrillation	27	4.3
PACs	7	1.1
SVT(AVNRT)	13	2.1
Wolf Parkinson White	4	0.6
Idioventricular rhythm	2	0.3
Ventricular tachycardia	2	0.3
PVCs	12	1.9

PACs, premature atrial contractions; SVT, supraventricular tachycardia; PVCs, premature ventricular complexes; AVB (atrioventricular block), LBBB & RBBB (left and right bundle branch block)

**Figure:**
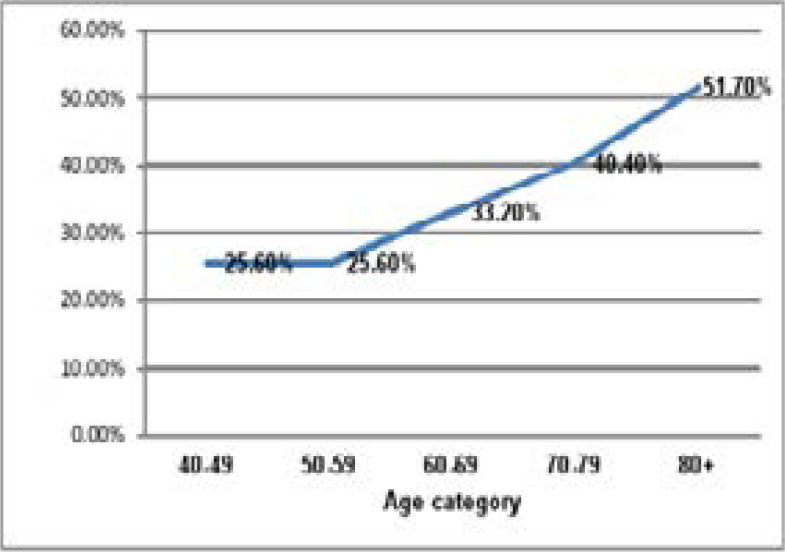
Shows prevalence of cardiac arrhythmias by age categories of study participants, Jimma Town, May to July, 2017.

### Socio-demographic and lifestyle risk factors of Arrhythmia

In multivariate analysis study participants who were smokers during the study, time was nearly 2 times more likely to experience arrhythmias than those who were not smokers (AOR=1.95; [95%C.I: 1.008, 3.760]; P=.047). Study participants who consumed solidified vegetable oil was more than 3 times more likely to have arrhythmia than compared with non-consumption of solidified vegetable oil (AOR=3.50;[95%C.I: 1.502, 8.175];P=.004).

Study participants who were retired, and who do not have a job (whose occupations were none) were 1.7 times more likely to have arrhythmia than employees (AOR= 1.78; [95%C.I: 1.108, 2.857]; p=.017). It was also observed that individuals who were involved in vigorous-intensity activities were protected by half from developing arrhythmias (AOR=.57; [95%C.I: .343, .929], P=.024) than those whose work doesn't involve vigorous-intensity activities. Study participants who were hypertensive were 1.5 times more likely to develop arrhythmia than those who were non-hypertensives (AOR= 1.56; [95%C.I: 1.047, 2.336]; P= .029). Study participants who had a history of MI were nearly 2 times more likely to have CA than those do not have MI (AOR-=1.78;[95%C.I: 1.029, 3.091], p=.039). Similarly, individuals who had congestive heart failure were 2.065 times more likely to develop arrhythmia than those who do not have congestive heart failure (AOR=2.07;[95%C.I: 1.103, 3.867];P=.023). In addition to this, participants who had a history of stroke were five times to have arrhythmia than those who did not have a history of stroke (AOR=4.98; [95%C.I: 1.921, 12.929]; P=.001). Participants were screened for obstructive sleep apnea (OSA) risk using the STOP questionnaire and accordingly, 311(49.1%) were low risk for OSA, and 323(50.9%) were high risk for OSA (See table 3).

**Table 3:** Multivariable logistic regression model predicting cardiac arrhythmias among study participants in Jimma Town from May to July 2017

Models	β	P	AOR	95% CI	
				
				Lower	Upper
Sex					
Male	.173	.468	1.188	.745	1.895
Female			1.000		
Age group(yrs)					
40–49			1.000		
50–59	-.253	.467	.776	.393	1.535
60–69	.021	.949	1.022	.534	1.954
70–79	.134	.698	1.144	.579	2.259
80+	.366	.383	1.443	.633	3.287
Occupation					
Employee		1.000		
Housewife	-.157	.586	.854	.485	1.505
Others (pensions, none)	.576	.017	1.779	1.108	2.857
Vigorous Physical Activity					
Yes	-.571	.024	.565	.343	.929
No			1.000		
History of myocardial infarction					
Yes	.579	.039	1.784	1.029	3.091
No			1.000		
Obstructive sleep apnea					
High risk	.272	.148	1.312	.908	1.895
Low risk			1.000		
Currently smoking					
Yes	.666	.047	1.947	1.008	3.760
No			1.000		
Congestive Heart Failure					
Yes	.725	.023	2.065	1.103	3.867
No			1.000		
History of Stroke					
Yes	1.606	.001	4.983	1.921	12.929
No			1.000		
Solidified vegetable oil consumption					
Yes	1.254	.004	3.504	1.502	8.175
No			1.000		
Hypertension					
Yes	.447	.029	1.564	1.047	2.336
No			1.000		

AOR=adjusted odds ratio; CI= confidence interval

(Hosmer Lemeshaw Test (P=0.982), Maximum Standard error=0.486)

## Discussion

Eighty percent of the global burden CVDs occur in low and middle-income countries(LMICs) and this high percentage is partly due to the much larger population in these countries, progress in avoidance of deaths from childhood diseases so that now more individuals live to older ages when they are at risk of developing CVDs. In addition to this increased tobacco use, decreased physical activities, increased use of animal products, and increased obesity with resultant elevations in blood pressure, cholesterol, and diabetes especially in the countries that are responsible for the burden^29^.

The best way to reduce the burden of CNCDs; especially cardiovascular diseases, such as cardiac arrhythmias, is early screening and taking preventive measures. In our study, the prevalence of cardiac arrhythmias and its associated factors in ambulant adult study participants of an urban Jimma community were examined. The result demonstrated that the prevalence of CA was 34.2%. In the literature, the reported incidence of arrhythmia in the community ranges from 14.9% to 59.1 %^11^–^13^. The explanation for this wide range includes the difference in characteristics of the population, the sample studied (e.g., age, lifestyle, type of screening tool used to detect arrhythmia), or study design employed.

In contrary to our study, a study conducted in Ontario, Canada found a prevalence of around 20% which is lower than the prevalence of the current study. The observed cardiac rhythm irregularities in this study took various forms: 8 ECGs displayed premature ventricular contraction, 3 had a premature atrial contraction, 2 had atrial fibrillation, 1 had missed beats and 1 had a bundle branch block which is different from the types of arrhythmias we found^14^. The possible reason for the differences in the prevalence of cardiac arrhythmias between the above study and our study might be the differences in the study design, sampling technique, sample size, and the screening tool (2-lead ECG) they used whose sensitivity and specificity was unknown which could not identify ‘true positives’ and ‘true negatives.

A study conducted by Özdemir et al.2012 among heavy vehicle drivers found a prevalence of 59.1% which is far more frequent than the prevalence that is found in the current study^13^. The discrepancy in the results might be due to the study period, and the characteristics of sampled participants enrolled in the study that heavy vehicle drivers spent much of their time driving sitting thus prone to a sedentary lifestyle which could accumulate their risk of obesity. The detailed mechanisms linking obesity and arrhythmia are complex and are indicated in the work of Yusuf S. et al^29^.

In the present study, the most common type of arrhythmia detected as conduction disturbances (sinus bradycardia 7.6%, first-degree atrioventricular block 1.1%, second-degree atrioventricular block 0.6%, third-degree atrioventricular block 0.2%, complete left bundle branch block 7.9%, incomplete left bundle branch block 1.7%, complete right bundle branch block 5.5%, incomplete right bundle branch block 0.8%; which account a total of 25.4%). More importantly, atrial fibrillation is the most frequently sustained arrhythmia with a prevalence of 4.3%.

This finding is consistent with a finding reported from a study conducted in the Chinese, Hong Kong community11. The current study was also in the same line with the study conducted on pre-hospital refer cases for syncope where ECG findings were; in 13% ECG showed sinus tachycardia, in 9% sinus bradycardia. Prevalence of ventricular tachycardia was 0.20%, while significant AV-disturbances were present in 1.12% of cases (0.11% second-degree type 2 atrioventricular block, 0.11% advanced atrioventricular block, 0.19% third-degree atrioventricular block, 0.45% junctional rhythm, 0.26% ventricular rhythm^30^. According to Brunetti ND et al aging affects the cardiovascular system in multiple ways, including a decrease in compliance of blood vessels through arterial stiffening and thickening, mild left ventricular thickening, and a shift in the balance of early versus late diastolic filling. Many of these changes result, in part, from cardiac cell enlargement with apoptosis of neighboring cells and subsequent fibrofatty infiltration of the myocardium. The conduction system of the heart is also affected by the latter, producing changes that may result in conduction disorders or arrhythmia^30^.

In the current study, atrial fibrillation was detected in twenty-seven participants who account for 4.3%. This result is comparable or in line with an institution and population-based study in some African countries 4.6% South Africa, 5.5% Ivory Coast, 5.4% Senegal, and more than study 0.7% Kenyan tertiary referral hospital(those patients might have taken medication for underlying cause), 0.7% in Tanzanian elderly(≥ 70yrs)^31^. In a study done in India on the prevalence of AF, they have found 5.1%; however, when compared with the current study it is different since they used a screening tool (Alivecor) whose sensitivity and specificity was unknown and their sample size is lower than the current study^32^. In a cohort study done in Malaysia, the prevalence of atrial fibrillation was found to be 0.54% which is lower than the current study the difference could be due to the study design and study period (44).

The current study is also inconsistent with a study done on the prevalence of atrial fibrillation in Gonder teaching hospital which was 28.7% 33. The reason for the discrepancy might be due to that the latter study was conducted among known stroke patients who were admitted in the hospital; not in the community.

The present study showed that CA varied with the occupational status of the participants with a higher proportion in pensioners and those who are jobless, which is in line with the study findings by Rodrigo J. et al in which case they found that patients with premature complexes were generally older and more likely to be retired^34^. Also, progressive increment in arrhythmia prevalence with age was noticed (it was 51.7% in those aged ≥80 years) and sex difference was observed when cross-tabulated by arrhythmia and occupation with male predominance. A similar study reported that men were found to have a 1.5-fold higher risk of developing an arrhythmia (AF) compared with women^35^.

Similarly, our study found that alcohol consumption has no significant association (P=0.947) with arrhythmia. In agreement with the current study, one study concluded that low levels of alcohol intake are not associated with the development of AF. In contrast to this study done in Germany confirmed there exists an association between sinus tachycardia and chronic alcohol consumption and atrial fibrillation in holiday heart syndrome^36^. The possible deviation in our study might be most of the study participants consume local liquors such as tella, tej, etc of which alcoholic content may be less than standard alcohol.

In our study smokers were nearly 2 times more likely to have arrhythmia than non-smokers. Alanna M. et al. also reported that smoking was associated with the incidence of arrhythmia, with more than a 2-fold increased risk of AF attributed to current smoking^37^,^38^.

In addition, the present study showed that khat chewing has no significant association with CA (p=0.652). In an argument with our study findings, a study conducted in Yemen in 2016 found that the prevalence of non-sustained ventricular tachycardia was higher among khat chewers^39^. The possible difference is that majority of the participants in our study were women (who do not culturally encourage to chew khat than men).

According to the present study, participants who were hypertensive are 1.5 times more likely to have arrhythmia than non-hypertensives. A similar study was done in Malaysia also found an association between CA and hypertension^40^. Other researchers reported a 3.46% prevalence in hypertensives, indicating hypertension as an independent predictor for commonest sustained arrhythmia^41^. Hypertension has two major consequences on the heart: left ventricular hypertrophy, and morphological and functional alterations of the coronary macro- and micro-vessels. These two cardiac modifications then cause 3 types of complications: myocardial ischemia left ventricular dysfunction and electrical instability, which are implicated in the pathogenesis of atrial and ventricular arrhythmias in hypertensive patients^42^.

The primary strength of the study is the use of hospital standard 12-leads ECG in a relatively sizeable sample in the general population and early screening of life-threatening arrhythmia to tackle morbidity and mortality that will ensue. However, it is likely that some participants with paroxysmal AF may have been missed. So, one of the limitations of the current study is our inability of using the Holter monitor ECG. In addition, we didn't examine the echocardiography recordings and so we were unable to see structural and dimensional views of the heart.

## Conclusion

The prevalence of CA in the current population is 34.2%. It increased with advancing age. The most frequent type of arrhythmia noted is conduction disturbance.

Independent predictors of CA in the population were smoking, occupation, hypertension, history of chronic cardiac diseases such as myocardial infarction, congestive heart failure, and prior stroke. However, the most potent predictor was a prior ischemic attack (stroke). Future research should look into further on the overall burden of CA using the Holter ECG monitor on a large-scale population.

## Abbreviations

BMI: Body Mass Index
CA: Cardiac Arrhythmia
CVDs: Cardiovascular Diseases
ECG/EKG: Electrocardiogram
LMICs: Low-and-Middle Income Countries
NCDs: Non-communicable Diseases
WHO: World Health Organization

## Declarations

### Availability of data and material

Data can be accessed from the corresponding author.
